# 

**Published:** 1897-10-30

**Authors:** 


					THE HOSPITAL. ABSOLUTELY PURE, therefore BEST. Oct. 30ih, is97.
CADBURY'S -at* W CADBURY'S
COCOA Mi flrl COCOA
P?"7"""""" NO ALKALIES USED.
0
tsnntr ? Wkbtcbh InfirhamY NaRf.OLK.AMB NORWICH HOSPI TAL
No. 579. Vol. XXIII. [SfSSSUO Saturday
Oct. 30th, 1897.
[Bubscnpnonarms,j pRICE TWOPENCE.

				

